# Cerebro-Spinal Meningitis, or Spotted Fever

**Published:** 1865-10

**Authors:** E. R. Travers

**Affiliations:** Amboy, Ills.


### CEREBRO-SPINAL MENINGITIS, OR SPOTTED FEVER.—TREATED BY BLISTERING AND BELLADONNA.

By E. R. TRAVERS, M. D., Amboy, Ills.

May 9th, 1865, I was sent for to see a patient, Miss Jannett Munger, aged eighteen years, residing at Mr. John Fagan's house, in Bureau county. I found her suffering from chronic spasmodic muscular contractions, with most distressing opistholonos, and partial paralysis of the limbs, and a little delirious, with several spots of a well marked florid color, with a quick but fluttering pulse, and moist white coating over the tongue, and considerable palpitation of the heart. After making some inquiries, I found that the day previous she had a well marked chill, with a feeling of general indisposition, which was followed by a high fever and soreness of the throat. Her bowels being constipated I ordered a mild laxative, and applied a blister over the back of the neck, and then had the blistered surface smeared with Ung. Belladonna. I also gave her the following mixture: Tr. Belladonna, Tr. Viratrum Viride, Tr. Digitalis, aa. m. iii. every hour, until she was easier, then I continued the same every three hours, and stopped the Belladonna as soon as I perceived its constitutional effects make their appearance. My patient improved under this treatment every day. Her spasms became less frequent, and of shorter duration. In seven days I pronounced her convalescent, and ordered her a preparation of Iron and Quinine, to take a couple of weeks. She is now quite well and entirely recovered. I would be pleased to learn if this remedy had ever been recommended for this disease before, or if any of your readers have tried it. I was led to adopt it from general principles, and I am persuaded to the opinion that in this case it proved a great desideratum.

